# Novel organoids and *ex vivo* models for advancing poultry coccidiosis research

**DOI:** 10.1017/S0031182025100668

**Published:** 2025-09

**Authors:** Phoebe Yuen Ka Chan, Bernat Marti-Garcia, Virginia Marugan-Hernandez

**Affiliations:** Department of Pathobiology and Population Sciences, The Royal Veterinary College, North Mymms, Hertfordshire, UK

**Keywords:** chicken coccidiosis, *Eimeria*, enteroids, *ex vivo*, intestinal explants, intestinal organoids, *in vitro*

## Abstract

*Eimeria* species, the causative agents of avian coccidiosis, are major pathogens in poultry, resulting in substantial economic losses and welfare concerns worldwide. Understanding their complex life cycle, including different developmental stages and host interactions, is essential for advancing control strategies. Traditional cultivation systems, such as primary cell cultures and immortalised cell lines, have provided valuable insights, but they present limitations in supporting complete parasite development, host–pathogen interactions and immune response evaluation. Recent advances in intestinal organoids offer a promising alternative for *Eimeria* research. Initially developed in human models, intestinal organoids have been successfully adapted to avian systems, replicating the architecture, cellular diversity and physiological functions of the chicken gut epithelium. These 3D models provide now a physiologically relevant platform for studying parasite development, host–pathogen interactions, immune responses and drug screening *in vitro*. Complementary tools, such as intestinal explants, could further enhance the experimental repertory available for investigating *Eimeria* species. Additionally, insights from studies on related apicomplexan parasites support the translational value of these systems. These innovative systems could support significant advances in *Eimeria* cultivation, enabling more robust and ethical research while reducing the use of experimental animals.

## Graphical abstract

**Figure:**
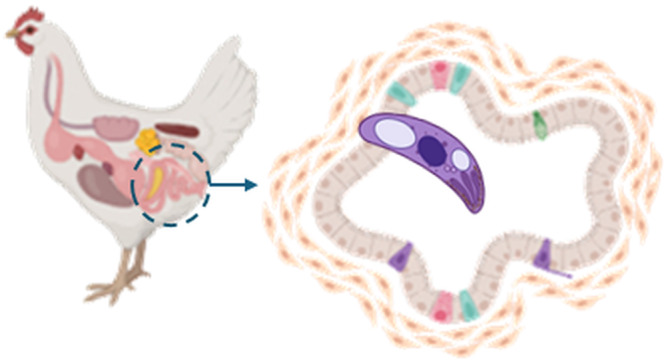

## Introduction

Chicken coccidiosis, caused by the protozoan parasite *Eimeria*, is an enteric disease that can occur in any type of production setting, from large commercial operations to small backyard flocks. It has wide-ranging impacts at the farm level, affecting not only the health of the birds but also the economic stability of poultry operations. Coccidiosis damages the intestinal lining of infected birds, which suffer from symptoms like diarrhoea, dehydration, weight loss and lethargy. These health issues reduce the overall health status of the flock, directly impacting on productivity and, in severe cases, infected chickens can die, leading to direct loss of stock. In addition to this, farmers often need to administer anticoccidial drugs or antibiotics to control the disease, leading to increased veterinary costs and growing problems of drug resistance, which result in reduced efficacy and more complex management programmes (Chapman and Rathinam, 2022).

Studying the avian intestinal tract and its pathogens, particularly *Eimeria* spp. protozoa, has been hindered by the lack of suitable, cost effective and reproducible cell culture models. One of the major challenges in studying coccidiosis is understanding how *Eimeria* interacts with the chicken intestinal tract at a molecular, cellular and tissue levels. Traditional animal models (live chickens) are valuable but can be expensive, can be time-consuming and may not always provide detailed insights into the specific molecular and cellular mechanisms of infection at cellular level.

**Figure 1.**
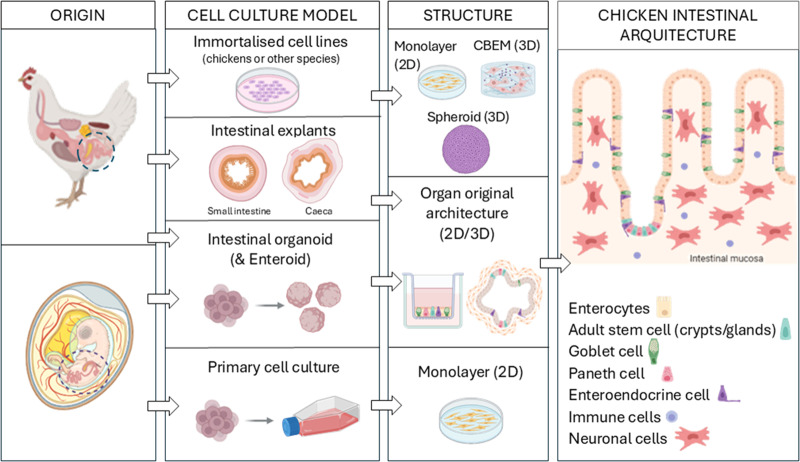
Existing and potential cell culture models for *Eimeria* spp. Cultivation. Cells or tissues can be extracted from adult chicken intestinal tissue or chicken embryos. These can then be used to develop various models, including primary cell cultures, immortalized cell lines, intestinal organoids or intestinal explants (Tables 1 and 2). Each of these models can be developed in 2D, 2.5D or 3D formats (scaffold-, spheroid- or organoid-based), with the most advanced models – organoids – closely resembling the architecture of the real chicken intestine. Figure created using BioRender.com.

The use of *in vitro* and *ex vivo* models for coccidiosis research (Table 1) offers a simplified and accurate way to study *Eimeria* infections and host–parasite interactions compared to animal models. In particular, chicken-derived intestinal organoids and enteroids (following the definition by Taelman et al., 2022) could provide an *in vitro* model that mimics the structure and function of the chicken intestine, offering a controlled environment for studying how *Eimeria* invades, modifies and damages intestinal cells. An overview of the different cell culture models already used or offering promise for *Eimeria* cultivation is provided in Figure 1 and Table 2.

**Table 1.** Definitions of existing and potential cell culture models for Eimeria spp. cultivation

Cell culture models	Description	Cell types	Experimental type
Primary cell cultures	Cells are derived from tissues of a live organisms and kept for short period of time	Single or small number of distinct cell types depending on the selection method	*In vitro*^a^
Immortalized cell lines	Cells can divide indefinitely due to genetic modifications	Single cell type	*In vitro*
Intestinal organoid	3D structures grown form stem cells that mimic the architecture and function of the intestine	Intestinal epithelial (enterocytes, goblet cells, Paneth cells and enteroendocrine cells) and non-epithelial cells (mesenchyme, immune cells)	*In vitro*
Enteroid	Subtype of intestinal organoid specifically derived from adult intestinal stem cells	Exclusively intestinal epithelial cells	*In vitro*
Intestinal explant	Piece of tissue that is removed from an organism and cultured outside the body	Intestinal epithelial (enterocytes, goblet cells, Paneth cells, enteroendocrine cells) and non-epithelial cells (mesenchyme and immune cells)	*Ex vivo*^b^/*in vitro*

a *In vitro*: System developed outside of a living organism.

b *Ex vivo*: Tissues or organs removed from a living organism that maintain their original architecture.

**Table 2.** Summary of existing and potential cell culture models for Eimeria spp. cultivation

Cell culture model	Cell line	Origin (cell type/organ)	Animal species	Infecting stage	Stage of development achieved	Reference
Primary cell culture	N/A	Epithelial/kidney	Chicken (embryo)	Sporozoite	Oocysts	Strout and Ouellette, 1969, Doran, 1971, Quist et al., 1993
Immortalized cell line	MDBK	Epithelial/kidney	Bovine	Sporozites	First-generation merozoites	Patton, 1965, Tierney and Mulcahy, 2003
Immortalized cell line (MC29 retrovirus transformed)	HD11	Macrophage/Blood	Chicken	Sporozoite	First-generation merozoites	Hériveau et al., 2000, Aguiar-Martins et al., 2024
Immortalized cell line (neoplastic)	LMH	Epithelial/liver	Chicken	Sporozoite	First-generation merozoites	Hériveau et al., 2000, Tierney and Mulcahy, 2003
Immortalized cell line	CLEC213	Epithelial/lung	Chicken	Sporozoite	First-generation merozoites and oocysts	Bussière et al., 2018
				Second-generation merozoite		
Immortalized cell line	BHK	Epithelial/kidney	Hamster	Sporozoite	First-generation merozoites	Tierney and Mulcahy, 2003
Immortalized cell line (neoplastic)	Caco-2	Epithelial/intestine	Human	Sporozoite	N/A	Tierney and Mulcahy, 2003
Immortalized cell line	DF-1	Fibroblast	Chicken (embryo)	Sporozoite	N/A	Liu et al., 2022
Immortalized cell line (SV40 large-T antigen transformed)	CIEC-H2	Epithelial/intestine	Chicken	Sporozoite	N/A	Ghiselli et al., 2023
Immortalized cell line (SV40 large-T antigen transformed)	AIEC	Epithelial/intestine	Chicken	Sporozoite	Second-generation merozoites	Chen et al., 2024
Immortalized cell line (neoplastic)	COLO-680N	Epithelial/oesophagus	Human	Sporozoite	First-generation merozoites	Aguiar-Martins et al., 2024
Immortalized cell line (neoplastic)	HCC4006	Epithelial/lung	Human	Sporozoite	First-generation merozoites	Aguiar-Martins et al., 2024
Intestinal organoid (basal-out)	N/A	Intestine	Chicken (embryo)	N/A	N/A	Pierzchalska et al., 2012
Enteroid (basal-out)	N/A	Intestine (caecum)	Chicken	N/A	N/A	Powell and Behnke, 2017
Enteroid (basal-out)	N/A	Intestine (jejunum)	Chicken	N/A	N/A	Li et al., 2018
Intestinal organoid (basal-out)	N/A	Intestine	Chicken (embryo)	N/A	N/A	Zhao et al., 2022
Enteroids (2D)	N/A	Epithelial/intestine	Chicken (embryo)	N/A	N/A	Orr et al., 2021
Intestinal organoid (basal-out)	N/A	Intestine	Chicken (embryo)	N/A	N/A	Oost et al., 2022
Enteroids (apical-out)	N/A	Intestine	Chicken	Sporozoites	Gametes	Nash et al., 2021,2023
Intestinal organoids (apical-out)	N/A	Intestine (embryo)	Chicken	N/A	N/A	Shin et al., 2025
Explants	N/A	Intestine (ileum)	Chicken	N/A	N/A	Zhang et al., 2017
Explants	N/A	Intestine (jejunum)	Chicken	N/A	N/A	Duarte et al., 2021
Explants	N/A	Intestine (ileum)	Chicken	N/A	N/A	Mátis et al., 2024, Mátis et al., 2025

Current developments in chicken organoid are limited to models including intestinal epithelial cells and not more complex systems, and their use for research in coccidiosis has been limited to study sporozoite infection and development. Future more complex organoid models could be used to observe immune cell recruitment, inflammatory responses and how the parasite modulates the host immune system in response to *Eimeria* infection. They could also serve as a valuable platform for testing anticoccidial drugs or new treatments prior to *in vivo* trials, allowing researchers to evaluate the effectiveness of various compounds in preventing or reducing infection. This approach offers a more cost-effective and controlled alternative to conventional *in vivo* studies, with potentially greater predictive value than current *in vitr*o models (Arias-Maroto et al., 2024).

Finally, organoids provide an ethical advantage by potentially reducing the need for live animal testing, which is particularly important in an era where animal welfare is a growing concern. By providing an alternative to some *in vivo* testing, organoids could help minimize the use of chickens in research, aligning with ethical and regulatory guidelines to promote the 3Rs (Replacement, Reduction, Refinement) in animal research.

In this review, we have extensively examined the literature on *in vitro* and *ex vivo* systems so far used for research in chicken coccidiosis (Table 2; Figure 1) and explored how these models, including intestinal organoids and explants, could benefit research in this problematic disease. Additionally, we reviewed recent advances in culturing related apicomplexan protozoa, such as *Cryptosporidium* spp. and *Toxoplasma gondii*, and discussed how these developments could inform research on *Eimeria*.

## Specific considerations for *Eimeria* species and their life cycle stages

**Figure 2.**
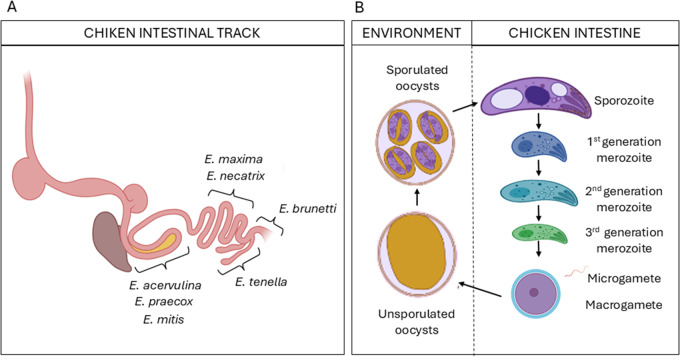
Sites of infection and specific stages of *Eimeria* spp. parasites. (A) Seven *Eimeria* species (*E. acervulina, E. brunetti, E. maxima, E. mitis, E. necatrix, E. praecox* and *E. tenella*) specifically infect distinct regions of the chicken intestinal tract. (B) In the environment, unsporulated oocysts become infective (sporulated oocysts) through the process of sporogony. Within the chicken intestinal tract, sporozoites are released from the oocysts and infect epithelial cells in the gut, where they undergo multiple rounds of schizogony and gametogony, producing different parasite stages at each round. Figure created using BioRender.com.

The parasite genus *Eimeria* comprises thousands of species that specifically infect a wide range of hosts, from mammals to reptiles, meaning that each parasite species infects only a single, specific host (monoxenous parasite). In chickens, 7 *Eimeria* species are recognized as causing coccidiosis (Burrell et al., 2020), with 3 additional cryptic species recently described whose implications are still under investigation (Blake et al., 2021). When studying and aiming to control coccidiosis, it is crucial to consider that the 7 *Eimeria* species affecting chickens infect distinct regions of the intestinal tract (Figure 2), each producing different pathological effects. The main molecular mechanisms underlying why each species targets a specific niche remain unknown (Lai et al., 2011), complicating the application of models designed to study these species concurrently.

The life cycle of *Eimeria* has been extensively reviewed elsewhere (Burrell et al., 2020; López-Osorio et al., 2020), so a detailed description is omitted here. Briefly, sporozoites excysted from oocysts, invade the host intestinal epithelium and develop into first-generation schizonts, which then progress to second-generation schizonts, and so on – typically through 3 rounds – before culminating in the sexual stages. A critical phase of the life cycle involves the fertilization of macrogametes by microgametes, leading to the formation of diploid zygotes. These zygotes mature into oocysts, which are essential for the faecal-oral transmission of the parasite between hosts. It is important to note that each replication cycle of the parasite results in the development of distinct stages (Figure 2), each exhibiting significant variations in morphology and gene expression, leading to distinct protein profiles (Sandholt et al., 2021). These differences complicate the characterization of host–parasite interactions.

Therefore, when aiming to develop novel *in vitro* models to study coccidiosis, it is essential to account for the distinct species and developmental stages of *Eimeria*. A ‘universal’ model may not be feasible, as each species exhibits unique intestinal region preferences and life cycle characteristics.

## Traditional and current cultivation models for *Eimeria* research

Cultivating *Eimeria* species *in vitro* has long been challenging due to their complex life cycles and host specificity. Traditional methods, such as embryo derived cells or primary chick kidney cells, have allowed partial reproduction of the *Eimeria* life cycle, but these models (2D) often yield low efficiency (Table 2). More recent advancements, including multilayered (3D) cell culture systems, have yielded promising results advancing our understanding of *Eimeria* biology and host–pathogen interactions, as they better reflect the micro-anatomical and physiological milieu (Figure 1). However, challenges remain in achieving full life cycle completion *in vitro*, and concerns about reproducibility and scalability must be addressed before these models can replace animal use in research. Among the 7 *Eimeria* species infecting chickens, *Eimeria tenella* has emerged as a model organism for the genus due to its enhanced adaptability to cultivation and genetic manipulation. Consequently, most recent studies developing *in vitro* and *ex vivo* models are based on this specific species.

### Primary cell cultures and immortalized cell lines (2D)

In the 1970s, the whole life cycle of *E. tenella* was achieved *in vitro* for the first time, evidenced by visual observation of oocysts, using a primary chicken kidney cell culture (Doran and Augustine, 1973). More recently, in 2010 the completion of the life cycle by presence of oocyst was reported again using caecal epithelial cells from chicken embryos in cell culture (Gu et al., 2010). Later, in 2018, Bussière et al. (2018) were able to observe gametes after infecting a lung chicken epithelial cell line (CLEC213) with second-generation merozoites. Other than in these studies, the complete biological life cycle of *Eimeria* with visual evidence of sexual stages or oocysts has not been successfully reproduced *in vitro* to the best of the authors’ knowledge.

Several immortalized cell lines have supported sporozoite invasion and partial development of *E. tenella* to first-generation schizonts (including bovine kidney cells (MDBK), human epithelial cells (Caco-2), chicken hepatoma cells (LMH), baby hamster kidney cells (BHK), chicken fibroblast cells (DF-1) and chicken macrophage-like cells (HD11) (Tierney and Mulcahy, 2003; Table 2). Interestingly, none of these cell lines originate from chicken intestine, the primary site of infection and reproduction for *Eimeria* spp., indicating reduced specificity of the parasite during the initial life cycle stages. Unlike primary cell cultures, immortalized cell lines can remain viable through multiple passages while maintaining their morphological and physiological characteristics. This capability provides a consistent supply of target cells, enhancing the reproducibility of experiments. Immortalization of avian cells is, however, inherently more challenging compared to mammalians ones, as avian species have naturally lower mutation rates.

Nonetheless, Ghiselli et al. (2023) and, more recently, Chen et al. (2024), have immortalized a primary cell culture originated from chicken intestinal epithelial cells using the SV40 large T antigen transduction methodology. Ghiselli et al. studies demonstrated the enterocyte nature of the cell line as these expressed markers and enzymatic activity of mature enterocytes. The ability of sporozoites to invade these cells was explored; however, the replicative capacity of *E. tenella* was not studied. Chen et al. model consisted in immortalization of epithelial that did not develop into enterocytes. When cells were infected with sporozoites, second-generation schizonts and merozoites were observed at 96 h post-infection, but not further stages. These 2 immortalized systems could be a more suitable model for high throughput anticoccidial drug screening trials. Nevertheless, the use of immortalized intestinal epithelial cell lines still presents several drawbacks, including a lack of cellular heterogeneity, partial reproduction of tissue functionality and the presence of genomic abnormalities, as previously discussed by Beaumont et al. (2021).

### Multidimensional cell culture models (3D)

As mentioned earlier, traditional culture systems, based on bidimensional cell monolayers (2D), have failed to replicate the complex cellular interactions present *in vivo*, limiting the completion of *Eimeria* life cycle. Alternatively, multidimensional (3D) culture systems are considered more physiologically relevant, offering better representation of the chicken intestinal microenvironment. These systems could facilitate more accurate studies of cell-to-cell and cell-to-matrix interactions, which are essential for understanding the biology and interactions of *E. tenella* with the intestinal mucosa, including the local innate immune system.

Aguiar-Martins et al.’s (2024) study has demonstrated the potential of adding collagen-based extracellular matrices (CBEM) to monolayered 2D cultures, enabling the multilayering (3D) of epithelial cells, for studying the growth and development of *E. tenella*. These culture systems have been trialled in multiple cell lines known to support *E. tenella* infection and replication, such as MDBK, HD11, and the human COLO-680N, and HCC4006 (Table 2), enabling parasite replication up to the first schizogony stage as early as 48 h post-infection. In this same study, scaffold-free cell spheroids (3D) were generated using HD11 cells, which showed lower viability rates compared to the CBEM system. Despite this, the spheroids still supported *E. tenella* invasion and the release of first-generation merozoites.

While these culture systems described here have supported invasion and development of early stages of *E. tenella*, they have not yet been able to replicate the full life cycle of the parasite. The progression to the sexual stages, including the development of gametocytes and oocysts, remains elusive in these models. Hence, further research is needed to develop more robust culture systems that can support the complete life cycle of *E. tenella*.

## Introduction to intestinal organoids

Intestinal organoids are 3-dimensional (3D) structures that can be generated by differentiation from embryonic pluripotent stem cells, induced pluripotent stem cells or adult intestinal epithelial stem cells (IESCs), the latter usually referred as ‘enteroids’ (Taelman et al., 2022; Table 1). Organoids offer a revolutionary platform for biological research (Sato et al., 2009; In et al., 2016) as they self-organize into complex structures that mimic the architecture, cellular diversity and physiological functions of the native intestine (Stelzner et al., 2012). They usually include various epithelial cell types such as enterocytes, goblet cells, Paneth cells and enteroendocrine cells, arranged in a polarized, villi-like configuration with crypt-like domain (Clevers, 2013). In contrast, 2D cultures restrict cell–cell interactions to lateral contact and typically lack spatial organization, extracellular matrix, cell polarity and dynamic microenvironments. These limitations can reduce their ability to replicate *in vivo* processes and may hinder accurate modelling of pathogen invasion and replication. Immortalized cell lines as those referred to in the previous section fail to replicate the epithelial complexity and innate immune responses essential for studying *Eimeria* infection dynamics within chicken intestine. Organoids have emerged to address these limitations, providing a more physiologically relevant model that simulates the intestine to study host–parasite interactions (Hares et al., 2021). By better mimicking intestinal structure and function, they serve as a bridge between conventional cell culture and whole-animal studies (Ramírez-Flores et al., 2022).

## Development of chicken intestinal organoids: from human to avian models

The journey of intestinal organoids began with human and mouse models. In 2009, Sato et al. (2009) established the first intestinal organoids from LGR5+ stem cells in mouse crypts, cultured in Matrigel with Wnt agonists (R-spondin 1), epidermal growth factor (EGF) and Noggin (WRN). Each of these components have essential roles in maintaining stemness and supporting the self-renewal of organoids by modulating key signalling pathways: Wnt antagonists will promote expansion of stem cells preventing premature differentiation, with R-spondin 1 exhibiting a synergistic role to amplify stem cell maintenance, with particular importance for LGR5+ stem cells, which are present in intestinal tissue; EGF is associated to intestinal proliferation; Noggin prevents differentiation into mature cell types by inhibiting Bone Morphogenetic Protein signalling. Organoids with budding crypts were able to produce and maintain stability for dozens of passages. These advancements laid the groundwork for species-specific adaptations in the generation of intestinal organoids (reviewed by Sato and Clevers, 2013).

Chicken intestinal organoids emerged later (reviewed by Nash and Vervelde, 2022), adapting mammalian protocols to avian physiology (Table 2, Figure 1). In 2012, Pierzchalska et al. developed the first chicken intestinal organoid from embryonic chicken intestines using a method previously developed for isolation of mice crypts and villi fractions (Barker et al., 2007), and medium containing EGF, R-spondin 1 and Noggin in Matrigel-coated plates. These organoids were ‘basal-out’ with the enclosed lumen, with crypt-like structure only occasionally seen. Similar results were obtained when Prostaglandin E2 was used instead R-spondin 1 and Noggin. It was 5 years later when Powell and Behnke (2017) generated enteroids for several animal species using the L-WRN cell line, which secrete the 3 WNR signalling factors (Wnt3a, R-spondin 3 and Noggin; Miyoshi and Stappenbeck, 2013), including an enteroid model derived from chicken caecal crypts, that were propagated up to 125 days. Soon after, in 2018, Li et al. developed chicken enteroids from small intestine. In this study, characteristic microvilli and crypts typical of mature enterocytes were observed by transmission electron microscopy (TEM), and the model seemed responsive to specific stimuli (CHIR99021, a glycogen synthase kinase 3β inhibitor), which supported comparable physiological function to the animal epithelium. Later, in 2022, Zhao et al. collected crypts from the small intestine of chicken embryos and adult chickens (layers and broilers). These crypts were then embedded in a basement membrane matrix and cultured with organoid growth media containing standard growth factors. The resulting organoids formed spherical structures with a polarized epithelium composed of enterocytes, where the presence of Paneth cells, goblet cells, fibroblasts and mesenchymal cells was confirmed by identification of specific markers by RT-PCR and immunoblotting.

Alternatively, Orr et al. (2021) developed a 2D enteroid model, comprising enterocytes, Paneth cells, goblet cells, enteroendocrine cells and leukocytes. Interestingly, this system self-organized into an exposed epithelial and mesenchymal sub-layer, and polarized into luminal, apical and basal aspects, mimicking more reliably the intestinal mucosa morphology (i.e. epithelial lining and lamina propria). Although *Eimeria* infections have not been assessed in this model, innate immune responses against lipopolysaccharide (LPS) from *Salmonella enterica* serotype Typhimurium were evidenced by an upregulation of inflammatory cytokines IL-6 and IL8. Additionally, the morphological and physiological properties of this system were preserved under freezing and thawing conditions.

Pierzchalska et al. (2017) studied for first time the effects of stimulation of Toll-like receptors (TLRs) of chicken intestinal organoids adding specific antagonists to TLR4 and TLR2 (types 1 and 2) or co-culturing them with *Lactobacillus acidophilus*. Interestingly, the TLR2 Pam3CSK4 and LA-5 ligand was able to modulate organoids by promoting growth and increasing prostaglandin (PGE_2_ and PGD_2_) levels. A major improvement came with studies (Oost et al., 2022), who discovered that supplementing cultures with chicken-derived RSPO1 and WNT3 (instead of mammalian ones), along with Prostaglandin E_2_ (PGE_2_) and a Forkhead box O1 (FOXO1) inhibitor, significantly enhanced their longevity (both in 2D and 3D cultures). Increased levels of PGE_2_ and PGD_2_ in longer-lived organoids had previously been observed by Pierzchalska et al. (2017), further supporting the role of prostaglandins in the survival of chicken intestinal organoids. These avian-specific factors stabilized stem cell signalling, extending organoid cultures to 15 passages and allowing the differentiation into different intestinal cell types (goblet cells, endocrine cells and enterocytes).

All these studies have supposed a major step forward, transforming chicken intestinal organoids from short-lived spheres into functionally relevant models.

## Advances in chicken organoids: towards *Eimeria* research

Early chicken intestinal organoids faced significant hurdles in infection studies. While Oost et al. (2022) developed stable cultures that grew as organoids in a gel-like protein matrix (i.e. Matrigel), these models were not tested for pathogen susceptibility. Additionally, the absence of immune system components limited their ability to mimic closer host–parasite interactions. Insights from human organoid research could help explain these challenges. In gel-embedded organoids, the basal side faces outward while the lumen remains enclosed; therefore, microinjection is usually required to introduce pathogens in human and livestock intestinal organoids (Heo et al., 2018; Beaumont et al., 2021). Alternatively, mechanical disruption or reversion to 2D cultures could expose the apical epithelium for parasite invasion (Derricott et al., 2019; Luu et al., 2019; Oost et al., 2022). These studies underscore the importance of organoid polarity in infection research. Shin et al. (2025) expanded on this concept by demonstrating how polarity – apical-out vs basal-out configurations – affects pathogen replication in chicken intestinal organoids. Using low-pathogenic avian influenza as a model, they showed that apical-out organoids, with lumens exposed, better mimic natural gut infections. In contrast, reversing polarity to expose basolateral surfaces altered the dynamics of infection.

Nash et al. (2021) marked a transformative shift by developing ‘inside-out’ chicken enteroids with an innate leukocyte component. By replacing traditional gel-embedded organoids to floating ones, these exposed their apical surfaces to the outside, in this way, pathogens could directly access epithelial targets, by simply adding them into the culture media, without supplementary techniques for inoculation. Caecal enteroids supported the infection by *E. tenella* sporozoites, revealing sporozoite invasion, intracellular replication (schizogony) and potential development into gametes supported by the presence of transcripts of *etgam56* gene. Validation of high level of reproducibility of this enteroid model came from Nash et al. (2023), who profiled floating apical-out chicken enteroids using temporal transcriptomics. Gene transcription demonstrated that these enteroids possessed the cellular diversity that is found in the chicken intestinal epithelium (Paneth cells, goblet cells, enterocytes, enteroendocrine cells, transit-amplifying cells, intestinal stem cells and tuft cells). There was also presence of transcripts for brush border enzymes and genes under the GO term ‘cell projection organisation’, which reflected microvilli development, which were further confirmed by TEM. There were also genes that represented several populations of lymphocytes (T cells, B cells, macrophages, dendritic cells and natural killer cells). One drawback of this model is the impossibility to propagate ‘apical-out’ floating enteroid cultures, unlike for gel-embedded ones, where continuous passage is possible. The reasons for this remain uncertain and not related to epithelial mesenchymal transitions or growth factor deficiencies.

Finally, it is important to consider that region-specific organoids may help refining infection models for each of the chicken *Eimeria* species, which infect different regions of the intestinal track. For example, Powell and Behnke (2017) developed cecum-derived organoids, which could better match *E. tenella*’s natural infection site, as Nash et al. (2021) demonstrated, suggesting that intestinal region-specific organoids could replicate infection patterns more accurately.

## Intestinal chicken explants

Intestinal explants also offer an alternative physiologically relevant *ex vivo* model for studying intestinal health, inflammation and host responses to external stimuli. Randall et al. (2011) reviewed the methods and applications of intestinal explants from humans and a few animal species, particularly for the study of inflammatory intestinal diseases, as well as their use in toxicology and drug development, with the potential to reduce animal use in human medicine. Explants are prepared by isolating small sections of intestinal tissue and maintaining them in culture conditions that preserve their native architecture. Different to cultures of cell monolayers, explants retain the full cellular complexity and spatial organization of the intestine, including polarized epithelial cells, lamina propria and resident immune populations. This structural integrity allows for a more accurate representation of tissue-level responses to microbial, dietary or environmental stimuli (reviewed by Russo et al., 2016).

Several studies have demonstrated the utility of chicken explants for short-term inflammatory modelling. Zhang et al. (2017) established a chicken ileal explant model to study responses to LPS, showing that explants remain over 90% viable for up to 2 h. LPS exposure induced nitric oxide release and upregulated inflammatory markers, confirming the tissue responsiveness and application for short-term studies. Similarly, Duarte et al. (2021) used jejunal explants in Ussing chambers to assess epithelial damage caused by deoxynivalenol, a mycotoxin commonly found in chicken feed. They reported histopathological changes including size reduction of enterocytes, denudation of villi and increased levels of apoptosis evidenced by accumulation of caspase-3. Application of an antimycotoxin additive ameliorated the deoxynivalenol harmful effects. This study demonstrated the explant model’s relevance for toxicological screening.

More recently, Mátis et al. (2024) used an ileal chicken explant to evaluate cathelicidin-2, as potential replacement to antibiotics treating intestinal infections. Cathelicidin-2 exhibited anti-inflammatory effects. A year later, these same researchers developed a miniature chicken ileal explant system using biopsy punches to optimize viability over time and experimental consistency (Mátis et al., 2025). Explants retained epithelial architecture and lining for at least 12 h and responded to stimulation with pathogen-associated molecular patterns, showing variations of expression levels for different cytokines. This validated the model for assessing intestinal innate immune signalling. The smaller explant format allowed medium-throughput screening while maintaining essential tissue-level features. Studies on explants infected with *Eimeria* have not been reported. However, our preliminary results (unpublished) demonstrate the ability of sporozoites to infect caecal explants generated by precision cut tissue slicing, as well as supporting parasite development in explants derived from previously *in vivo*-infected caeca. Nevertheless, completion of the life cycle evidenced by visualization of oocysts was not achieved.

In comparison to explants, intestinal organoids require extended culture and external signals to reach differentiation. While organoids offer advantages in scalability, long-term culture and genetic manipulation, they typically lack immune components unless co-cultured and require polarity modification for pathogen access (Nash and Vervelde, 2022). Explants, by contrast, retain luminal orientation and immune elements natively, making them more suited for rapid, physiologically grounded assessments. Therefore, organoids and explants could serve complementary roles depending on the experimental timescale and complexity required.

## Development of cell culture and intestinal organoids in related apicomplexa

There are *Eimeria* closely related apicomplexan parasites for which cell cultures and organoid models have been used to study the invasion and replication (Hares et al., 2021) and that this review examined for their potential application to coccidiosis research.

Cryptosporidiosis is a major cause of diarrheal disease, particularly in young children, immunocompromized individuals and neonatal ruminants. Similarly to *Eimeria* spp., understanding the pathogenesis of *Cryptosporidium* spp., monoxenus parasites, has been historically hindered by the absence of physiologically relevant *in vitro* models that replicate the complex architecture of the small intestinal epithelium. Conventional cell lines, such as HCT-8 and Caco-2, derived from human intestinal carcinoma cells, have supported partial parasite development but fail to recapitulate the complete life cycle or accurately mimic the intestinal niche (Yu et al., 2000). However, in 2016, Morada et al. (2016) reported that the adaptation of hollow fibre technology using HCT-8 cell line allowed the continuous production of oocysts *Cryptosporidium parvum* for 6 months. In 2019, Wilke et al. (2019) described the use of an air–liquid interface (ALI) cultivation system based on IESC derived (Wang et al., 2015) to allow the completion of the life cycle and long-term cultivation of *C. parvum*, providing an accessible model to study the biology and host–parasite interactions. Application of organoid systems derived from human LGR5+ intestinal stem cells has also enabled completion of the life cycle and oocyst production (DeCicco, RePass et al., 2017). These systems have allowed to expand host–pathogen interaction studies under more *in vivo*-like conditions (reviewed by Bhalchandra et al., 2020; Marzook and Sateriale, 2020). Techniques such as microinjection and apical surface exposure – achieved through organoid fragmentation followed by monolayer culture – have facilitated infection and life cycle completion in these models, as well as in lung organoids (Heo et al., 2018). Additionally, murine colonic explants were developed by Baydoun et al. (2017) as an *ex vivo* model for studying cryptosporidiosis.

In contrast to *Eimeria* spp. and *Cryptosporidium* spp., *T. gondii* exhibits broader host range and tissue tropism, infecting most warm-blooded animals and a wide range of cell types, with a particular affinity for muscle and nervous tissues, with infections starting though the oral route. However, the development of asexual intestinal and sexual stages is restricted to feline intestines (where the parasite develops a life cycle equivalent to *Eimeria* and *Cryptosporidium*), and studies on these stages have been neglected compared to studies in tachyzoites and bradyzoites due to the lack of suitable models for cultivation. Classic *in vitro* models, including feline epithelial intestinal cells, as well as murine and human immortalized cell lines such as Caco-2, SW480 and T84, have supported advances in understanding invasion and replication mechanisms in the intestine; however, they lack the tissue complexity necessary to fully mimic *in vivo* conditions (Sena et al., 2023). Recent advances include the development of 3D intestinal organoids can offer more spatially and physiologically relevant platforms for studying host–pathogen intestinal interactions (Luu et al., 2019). Intestinal organoid models facilitate early-stage infection studies and enable investigation of animal species-specific responses (reviewed by Hares et al., 2021; Sena et al., 2023). Regarding the specific cat intestinal stages, murine-derived organoids treated with a delta-6-desaturase inhibitor to mimic feline intestinal environments supported sexual development *in vitro* (Martorelli Di Genova et al., 2019). Importantly, the transcription factors leading the transition from tachyzoite to merozoites have recently been identified (Antunes et al., 2024), which can significantly help to studies in these stages without relying on cats to obtain oocysts to release sporozoites to commence infections. Interestingly, a human intestine-on-a-chip model has recently been developed by Humayun et al. (2022), incorporating a microfluidic system that simulates local vascularization. Infections of this model with *T. gondii* have allowed the study of specific immunoresponses and immunomodulatory roles of specific cytokines.

## Conclusions and future directions

Cultivation of apicomplexan parasites has been crucial over the past 50 years for advancing our understanding of their biology and molecular mechanism of important processes such as host cell invasion and replication (Feix et al., 2023). In particular, the ability to maintain early stages of *Eimeria* spp. (i.e. sporozoites and first-generation schizonts) within host cells has enabled the detailed study of these molecular mechanisms for these specific parasites (Marugan-Hernandez et al., 2021; Burrell et al., 2022). This *in vitro* cultivation has also facilitated the development of pre-screening assays for identifying novel compounds with anticoccidial activity against *Eimeria* (Thabet et al., 2017; Arias-Maroto et al., 2024). However, despite the promising results from the 1970s regarding the observation of sexual stages and oocyst production in cell culture using primary cell cultures (Table 2), there has been little to no progress in this area since. *Eimeria* first-generation merozoites appear to be reluctant to reinvade host cell, not only non-chicken cells but also chicken-derived cell lines. This limitation prevents progression to the second-generation and further stages, which is critical for completing the life cycle. This reinvasion failure represents a major bottleneck in *Eimeria in vitro* cultivation.

Recent advancements in cell culture models, particularly the development of immortalized avian epithelial cell lines (Ghiselli et al., 2023; Chen et al., 2024) and the use of 3D culture models based on immortalized lines (scaffold- or spheroid-based; Aguiar-Martins et al., 2024), initially offered promise as they provide a more accurate representation of *in vivo* conditions. However, despite these improvements, the parasite appears to behave similarly to how it does in traditional 2D culture systems, halting development at the stage of first-generation merozoites. Therefore, important challenges remain to be addressed, particularly the ability to support the parasite’s complete life cycle *in vitro*.

In the past decade, there has been significant progress in the development of ‘basal-out’ chicken intestinal organoids (Table 2), providing improved culture conditions, enhanced differentiation and extended lifespans. These developments culminated in an ‘apical-out’ model with significant potential for studying avian infectious diseases, including *Eimeria* spp. (Nash et al., 2021). Although research on *Eimeria* infection in chicken organoids is still in its early stages, initial findings are promising. Nash et al. (2021) provided the most compelling evidence to date, demonstrating that inside-out enteroids can successfully support *E. tenella* infection and intracellular replication. In our opinion, this model represents a critical step towards the development of physiologically relevant *Eimeria* infection systems.

Adapting these advanced systems for *Eimeria* cultivation holds great potential for future breakthroughs. Such models could enable the study of previously neglected life cycle stages, such as merozoites and sexual stages, which play crucial roles in disease pathogenesis but remain poorly studies, even in other relevant Apicomplexa. Examining cultivation systems for the intestinal and sexual stages of *Cryptosporidium* spp., the adaptation of the ALI cultivation system (Wang et al., 2015) to chicken enteroids could be of interest for coccidiosis research. Moreover, the application of hollow fibre technology to 2D chicken enteroid models (Orr et al., 2021) could support the scaling up of these organoid systems to produce oocysts *in vitro*, potentially reducing the large number of chickens currently required for oocyst production in vaccine manufacturing. Advancing research on the intestinal and sexual stages of *T. gondii* is limited by the parasite’s definitive host, the cat. Research involving this species is typically subject to strict regulations, and both oocyst production and the development of cat intestinal organoids from feline tissues rely on live animals. In this context, significant progress has been made in manipulating tachyzoites to differentiate into merozoites, thereby bypassing the oocyst/sporozoite stage (Antunes et al., 2024). Additionally, efforts have been made to ‘felinise’ mice and mouse-derived cells to render them susceptible to sporozoite and merozoite infections (Martorelli Di Genova et al., 2019).

Looking ahead, chicken intestinal organoids hold strong potential for drug screening, providing a gut-like environment for testing novel compounds, as well as for vaccine development, enabling the assessment of antigen responses in immune-enhanced organoids (Mitchell et al., 2024). However, current models still lack adaptive immune components, which limits their ability to accurately replicate infections and systemic host responses (Lahree and Gilbert, 2024). Future advancements could focus on integrating microbiota or microfluidic systems to better replicate the gut ecosystem, addressing these limitations and further enhancing the model’s physiological relevance (Holthaus et al., 2021).
